# Gut microbiome in sepsis: from dysbiotic biomarker to precision and palliative decision-making

**DOI:** 10.3389/fmed.2026.1811304

**Published:** 2026-04-13

**Authors:** Li-min Wang, Chu Chen, Jian-cuo Danzheng, Jing Zhao

**Affiliations:** 1 Department of Clinical Laboratory, Shaanxi Province Hospital of Traditional Chinese Medicine, Xi’an, China; 2 Department of Clinical Laboratory, Shaanxi Provincial People’s Hospital, Xi’an, China

**Keywords:** biomarkers, dysbiosis, gut microbiome, immunometabolism, microbiome therapeutics, precision medicine, sepsis

## Abstract

Sepsis is a major cause of mortality in critically ill patients, necessitating improved early detection, risk stratification, and individualized clinical decision-making. The gut microbiome actively regulates host immunity, metabolism, and barrier function, engaging in bidirectional interactions with sepsis progression. Evidence suggests that gut dysbiosis not only accompanies sepsis but may also accelerate it. Characteristic shifts, including reduced microbial diversity, expansion of opportunistic pathogens, and decreased short-chain fatty acid production, could offer early prognostic signals prior to clinical decline. Advances in multi-omics and computational analytics are enabling the translation of microbial signatures into actionable clinical insights, supporting phenotype-specific stratification in sepsis. Emerging microbiome-targeted interventions such as next-generation probiotics, synbiotics, metabolite supplementation, and fecal microbiota transplantation show potential for modulating host responses in a stage-specific manner. Within a precision medicine framework, microbiome-derived biomarkers may refine both critical care management and palliative decision-making. In advanced or refractory sepsis, these insights could help tailor treatment intensity, prioritize symptom control, and avoid non-beneficial therapeutic escalation. Realizing this potential will require prospective validation and standardized approaches to integrate microbiome data into personalized, goal-concordant sepsis care.

## 1 Introduction

### 1.1 Clinical challenge of sepsis

Sepsis is a leading cause of global mortality, characterized by high incidence, high fatality rates, and significant clinical heterogeneity (1). Its pathogenesis involves a life-threatening dysregulated host response to infection (2). Current reliance on conventional biomarkers, such as procalcitonin and C-reactive protein, is limited by their lack of specificity and sensitivity for early detection (3). This often results in delayed diagnosis and intervention, contributing to poor outcomes. Therefore, identifying novel biomarkers capable of early and accurate prediction is a critical unmet need in sepsis management (4).

### 1.2 Central role of gut microbiome in systemic inflammation and immunity

The gut microbiome, a complex microbial community, plays a fundamental role in maintaining systemic immune homeostasis and modulating inflammatory pathways (5). Through the conceptual framework of the “gut-organ axis,” bidirectional communication between the microbiome and distant organs is well-established. In sepsis, this relationship becomes pivotal. The systemic insult of sepsis can induce intestinal barrier disruption and microbial dysbiosis (6). Conversely, the gut microbiome and its metabolites actively regulate remote organ inflammation and immune function (7). This creates a vicious cycle, where sepsis disrupts gut ecology, which in turn exacerbates systemic disease progression (8).

### 1.3 Limitations of current biomarkers and imperative for precision medicine

Existing biomarkers primarily reflect downstream inflammatory events and fail to capture the underlying biological heterogeneity of sepsis patients (9–11). This limits their utility for early warning, precise phenotyping, and personalized prognosis. The paradigm of precision medicine necessitates a more comprehensive approach (9, 10). Integrating the gut microbiome—a key modulator of host physiology—into sepsis biomarker panels offers a promising strategy (12). Assessing microbial composition, function, and metabolic output could provide a dynamic, systems-level view of the host response, enabling more accurate risk stratification and tailored therapeutic strategies (13).

In parallel with these developments, palliative care has emerged as an increasingly integral component of sepsis management, particularly in advanced or refractory cases (14). Clinical decisions regarding the escalation, de-escalation, or limitation of life-sustaining treatments are frequently made under conditions of significant uncertainty, where conventional physiological indicators may be insufficient to capture the reversibility of disease processes (15). In this context, biologically informed tools—such as microbiome-derived biomarkers—may offer additional insights into host resilience, immunometabolic reserve, and the likelihood of recovery (16, 17). Such information has the potential to support more nuanced, goal-concordant decision-making, including the timely transition from life-prolonging interventions to comfort-focused care when appropriate (18).

### 1.4 Objectives of this article

This perspective article aims to evaluate the potential of the gut microbiome as a dynamic biomarker suite for sepsis. We will scrutinize evidence on microbial dysbiosis patterns associated with disease onset, progression, and outcomes. Furthermore, we will explore the translational pathway from microbial profiling to clinically actionable tools, discussing challenges and future directions for developing microbiome-based diagnostics and personalized interventions, such as targeted microbial modulation.

**Figure 1:**
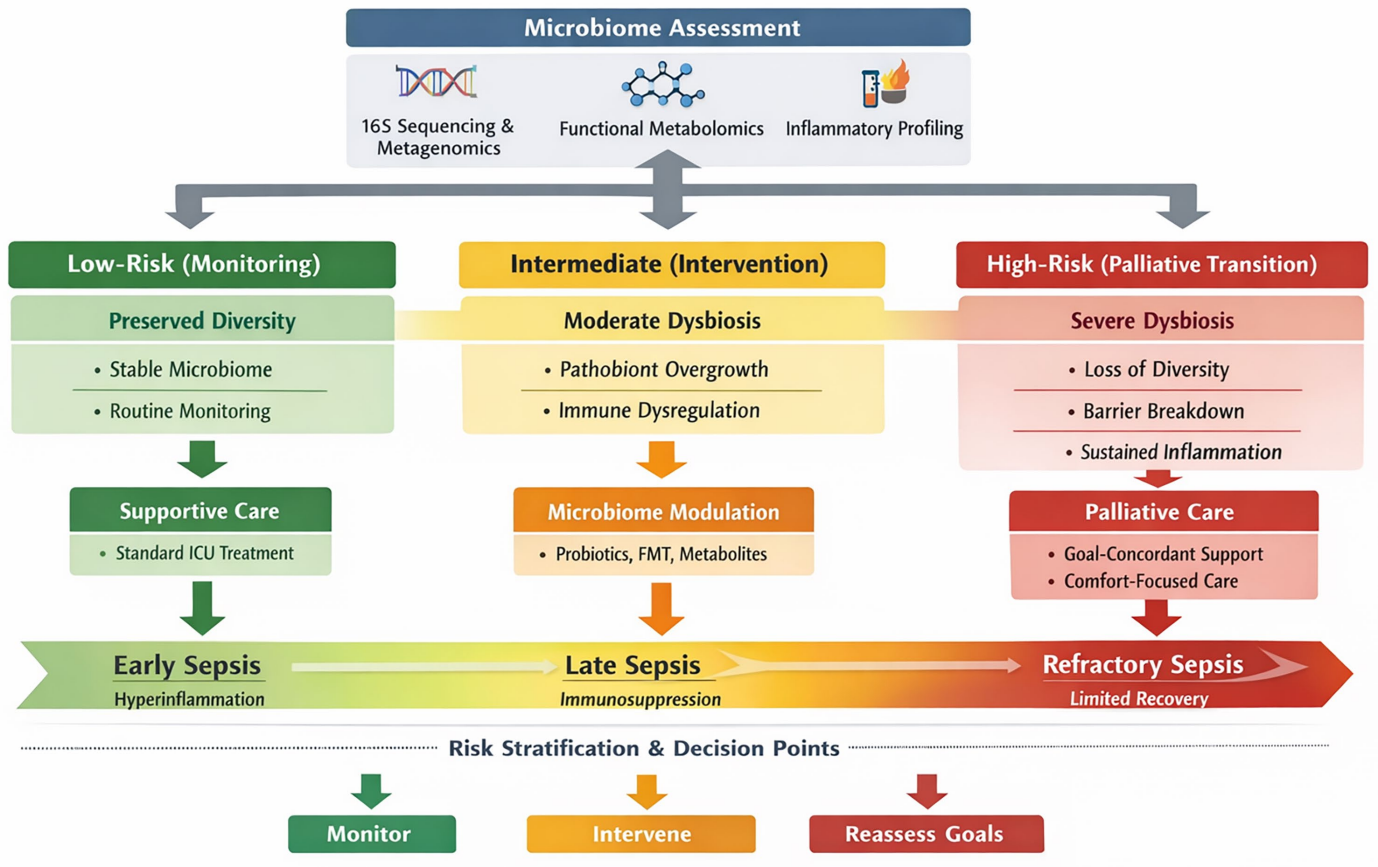
Microbiome-guided translational pathway for precision stratification and palliative decision-making in sepsis.

The overall translational framework is illustrated in Figure 1, providing a structured and clinically oriented overview of how microbiome-derived information may be integrated into real-world clinical workflows. To enhance clinical interpretability, Figure 1 explicitly illustrates microbiome-derived profiles to corresponding clinical decision pathways by categorizing patients into three biologically and clinically meaningful strata: preserved microbial diversity (reflecting low risk and potential reversibility), intermediate dysbiosis (indicating a potentially intervention-responsive state), and severe or persistent dysbiosis (suggesting limited reversibility and possible progression toward irreversible immunometabolic dysfunction). Each stratum is aligned with a distinct clinical strategy—ranging from monitoring and supportive care, to targeted microbiome modulation, and ultimately to consideration of treatment de-escalation and transition to palliative care—thereby transforming microbiome data from descriptive biomarkers into actionable decision-support signals.

To advance beyond descriptive and prognostic applications, we propose a novel conceptual framework—the Microbiome-Guided Decision Continuum (MGDC). This framework embeds microbiome-derived data into a three-dimensional clinical decision-making architecture comprising: (1) biological stratification based on microbial endotypes, (2) temporal staging of sepsis progression, and (3) alignment with goal-concordant care, including transitions toward palliative strategies (12, 13, 19). Distinct from prior models that primarily emphasize diagnosis or prognosis, the MGDC framework underscores the dynamic utility of microbiome information in guiding treatment intensity across the entire disease trajectory, from early intervention to end-of-life care.

Additionally, this perspective article extends its scope beyond conventional endpoints such as early detection and prognostication, to investigate how microbiome-derived biomarkers may inform precision decision-making within palliative care contexts in sepsis. Specifically, we examine the potential of gut microbial signatures to guide tailored treatment intensity, optimize symptom-based interventions, and support decisions to avoid non-beneficial therapeutic escalation in patients with advanced or refractory disease.

## 2 Pathophysiological link between gut microbial dysbiosis and sepsis

### 2.1 Dysbiosis as an “accelerator” of sepsis

Gut microbial dysbiosis is not only a consequence of sepsis but acts as a critical accelerator of its pathology. This role operates through three interrelated mechanisms. First, the structure of the gut microbiota is significantly altered: beneficial commensals like Bifidobacterium and Lactobacillus decline sharply, while potential pathogens such as Enterobacteriaceae expand (17, 20). This reduces microbial diversity and destabilizes the ecological community. Second, this imbalance directly compromises intestinal barrier integrity. The loss of commensals thins the mucus layer and downregulates tight junction proteins, increasing intestinal permeability (21). Concurrently, production of key microbial metabolites—particularly short-chain fatty acids (SCFAs) like butyrate—is markedly diminished (19). SCFAs are vital both as an energy source for epithelial cells and as immunomodulatory molecules. Their deficiency further weakens the barrier and disrupts immune regulation, for example by impairing regulatory T cell function (22). Finally, these changes enable bacteria and their products (e.g., lipopolysaccharide) to translocate into systemic circulation. This triggers and sustains a systemic inflammatory response, creating a vicious cycle of “barrier disruption → translocation → inflammation → further barrier damage” that exacerbates multi-organ dysfunction in sepsis (17, 19).

### 2.2 Microbiome and immunometabolic reprogramming

The gut microbiome critically influences the immunometabolic reprogramming characteristic of sepsis. Under homeostasis, microbial signals help “train” innate immunity and maintain tolerance (23). In sepsis, severe dysbiosis disrupts this balance. Loss of microbial diversity further disrupts immune tolerance mechanisms, potentially predisposing the host to uncontrolled inflammation and cytokine storm (19, 24). Furthermore, distinct shifts in the microbiome correlate with different clinical phases of sepsis. Early hyperinflammation is often associated with expansion of Gram-negative pathobionts, which may perpetuate inflammation via Toll-like receptor activation (25). In contrast, the late immunosuppressive phase coincides with profound microbial depletion and loss of metabolic function, which may be linked to immune cell exhaustion and increased susceptibility to secondary infections (26). Monitoring these dynamic microbial changes could therefore offer novel insights into a patient’s immunological status and guide phase-specific therapies (19).

## 3 Evidence for gut microbiome as a predictive and prognostic biomarker in sepsis

Building upon the mechanistic framework, subsequent sections focus on translating microbiome alterations into clinically actionable biomarkers and therapeutic strategies.

### 3.1 Predictive biomarkers

Emerging evidence supports the role of gut microbiome alterations as early indicators of sepsis risk (27). Key categories of microbiome-derived biomarkers, their biological implications, and clinical applications are summarized in Table 1. Quantifiable metrics, such as dysbiosis indices derived from microbial community structure, exhibit significant changes in critically ill patients prior to clinical sepsis onset (27). Notably, an elevated Enterobacteriaceae-to- Bacteroidaceae ratio is consistently linked to subsequent sepsis development, potentially signifying a pro-inflammatory shift in the gut environment (28). Beyond taxonomic composition, functional metagenomic analyses reveal that diminished functional capacity of the microbiome correlates with impaired immune defense and heightened infection susceptibility (29). This functional dysregulation may precede overt symptoms, offering a pre-clinical window for risk stratification (30).

**Table 1:** Gut microbiome–derived biomarkers in sepsis: clinical relevance and applications.

Biomarker category	Representative features	Biological relevance	Clinical utility	Disease stage
Taxonomic shifts	↓ diversity, ↑ Enterobacteriaceae	Pro-inflammatory bias	Early risk prediction	Pre-sepsis/early sepsis
Functional capacity	↓ SCFA synthesis genes	Barrier and immune dysfunction	Prognosis	Acute sepsis
Metabolites	↓ butyrate, bile acids	Immunometabolic modulation	Phase stratification	Hyper−/immunosuppressive
Integrated signatures	Multi-omics + AI	Systems-level phenotype	Decision support	ICU/palliative

SCFA, short-chain fatty acids; AI, artificial intelligence; ICU, intensive care unit.

However, it should be noted that much of the current evidence supporting microbiome-based predictive biomarkers is derived from observational studies with relatively small sample sizes and heterogeneous clinical populations (31). These methodological constraints may introduce bias, limit reproducibility, and restrict the generalizability of findings across diverse clinical settings (32). Consequently, while these biomarkers demonstrate promising predictive potential, their clinical applicability remains provisional and requires further validation through large-scale, well-designed prospective cohort studies.

### 3.2 Prognostic biomarkers

Longitudinal changes in the gut microbiome correlate strongly with clinical outcomes (33). Survivors typically show microbial resilience, characterized by recovering diversity and restoration of beneficial taxa such as Lachnospiraceae, which parallels organ function recovery and improved survival (33). In contrast, persistent dysbiosis independently predicts adverse outcomes (34). This microbial state is associated with prolonged immunosuppression and increased risk of secondary infections, providing a biological basis for prognostic assessment and guiding intervention timing (30, 35).

Despite these clinically relevant associations, the current evidence base remains largely derived from observational studies with limited methodological standardization (36). Variability in study design, patient populations, and analytical approaches constrains the reproducibility and generalizability of findings (37, 38). Furthermore, the causal relationship between microbiome dynamics and clinical outcomes has not yet been definitively established, making it difficult to distinguish whether observed microbial alterations represent drivers of disease progression or secondary epiphenomena (36, 39). Therefore, robust prospective validation and standardized study frameworks are essential before microbiome-based prognostic biomarkers can be reliably integrated into routine clinical practice.

### 3.3 Technological platforms and data integration

Advancements in multi-omics enable robust microbiome biomarker development. 16S rRNA sequencing profiles microbial composition, while shotgun metagenomics details functional potential and resistance genes (40, 41). Metabolomics quantifies microbiome-derived metabolites in biological samples, linking microbial activity to host physiology (42). Integrating these data layers offers a systems-level view of host-microbiome interactions (42). Artificial intelligence and machine learning are critical for analyzing these complex datasets, identifying biomarker patterns, and building predictive models that may outperform traditional clinical tools, thereby supporting personalized management strategies (43).

Despite these technological advances, several critical limitations continue to impede clinical translation. First, the current evidence base remains predominantly observational, with a paucity of well-designed prospective and interventional studies, thereby limiting causal inference (36). Second, the generalizability of findings is constrained by small sample sizes, substantial inter-individual variability, and heterogeneity in patient populations, clinical settings, and disease stages (31, 37, 38). Third, methodological inconsistencies—particularly in sample collection, processing, sequencing platforms, and bioinformatic pipelines—further compromise reproducibility across studies (37). Importantly, the causal relationship between microbiome alterations and sepsis outcomes has not yet been firmly established, making it challenging to distinguish true pathogenic drivers from secondary epiphenomena or treatment-related effects (44). Moreover, caution is warranted when interpreting findings from recent studies, as many remain preliminary and lack external validation (45). This highlights the necessity for replication in independent cohorts and emphasizes the importance of robust validation frameworks before translating emerging microbiome signatures into clinically actionable tools (45). Collectively, these limitations underscore the need for standardized protocols, longitudinal study designs, and mechanistic investigations to support the robust clinical implementation of microbiome-informed precision strategies.

## 4 From biomarkers to intervention: advancing toward personalized microbiome modulation

To establish a clearer translational framework, we propose an operational model that integrates microbiome-derived data into staged clinical decision-making. This model comprises three core components: (1) risk stratification based on the severity of microbiome disruption, (2) alignment with the prevailing disease phase—hyperinflammatory, immunosuppressive, or recovery—and (3) corresponding escalation or de-escalation of intervention intensity. Within this framework, microbiome profiles function as dynamic biomarkers that inform transitions across monitoring, active treatment, and palliative-oriented care.

### 4.1 Precision stratification of intervention strategies

Personalized microbiome interventions should be tailored to a patient’s risk profile and clinical stage. For high-risk populations such as intensive care unit patients, an early baseline assessment of the gut microbiome upon admission can inform preventive measures (18). Identifying individuals with significant baseline dysbiosis or depletion of beneficial taxa allows for targeted interventions aimed at stabilizing dysbiosis patterns identified at baseline, potentially reducing sepsis susceptibility (35). During sepsis, interventions should be phase-specific. In the early hyperinflammatory stage, strategies should focus on curbing pathogen expansion and supporting commensal colonization to temper excessive inflammation (19, 25). In later immunosuppressive or recovery phases, the priority shifts to restoring clinically favorable microbiome profiles to reduce secondary infection risk and facilitate organ recovery (19, 46).

From a practical perspective, microbiome-informed risk stratification can be organized into three actionable tiers (Figure 1). The first is a low-risk profile, characterized by relatively preserved microbial diversity and functional capacity, where routine monitoring and supportive care are appropriate (47). The second is intermediate-risk dysbiosis, marked by reduced diversity and the expansion of opportunistic taxa; in this group, targeted microbiome modulation may offer clinical benefit (48). The third is high-risk or severe dysbiosis, defined by profound microbial depletion and functional collapse, which may signal limited reversibility and prompt consideration of treatment limitation or a transition to palliative care (34). While these categories remain conceptual at present, they offer a clinically interpretable framework to guide future validation efforts.

### 4.2 Innovations and evidence in intervention modalities

Interventions are evolving toward more precise, function-targeted approaches. Next-generation probiotics include selected strains with defined immunomodulatory or metabolic functions, such as butyrate production (49). Synbiotics—combining probiotics with tailored prebiotics—enhance the engraftment and activity of beneficial microbes (50). Direct supplementation of key microbial metabolites, like butyrate or secondary bile acids, offers a direct route to modulate host physiology without relying on bacterial colonization (51). Meanwhile, traditional approaches such as selective digestive decontamination require reevaluation through a microbiome lens, weighing infection control benefits against potential ecological disruption (52). For recurrent or multidrug-resistant sepsis, fecal microbiota transplantation (FMT) is under investigation as a strategy to reintroduce a resilient, diverse microbial community (53).

Despite these advances, the current clinical evidence supporting microbiome-targeted interventions remains heterogeneous and, in some instances, conflicting (54). While meta-analyses suggest that probiotics may reduce infection rates in critically ill populations, concerns persist regarding their safety—particularly the risk of probiotic-associated bloodstream infections in immunocompromised or severely ill patients (55). Similarly, although FMT has demonstrated therapeutic potential in restoring microbial diversity, robust evidence from well-designed randomized controlled trials in sepsis populations remains limited (56). Additionally, many intervention studies are constrained by methodological limitations, including small sample sizes, heterogeneity in treatment protocols, and relatively short follow-up durations (57). These factors contribute to variability in reported outcomes and reduce the overall strength and reproducibility of the evidence base (58).

Collectively, these uncertainties necessitate cautious interpretation of existing findings and highlight the need for rigorously designed, adequately powered prospective studies (59). Future research should prioritize standardized intervention protocols, longer follow-up periods, and multicenter randomized controlled trials to establish both efficacy and safety across diverse clinical settings, thereby facilitating the translation of microbiome-based therapies into evidence-based clinical practice.

### 4.3 Special considerations in palliative care settings

In palliative care for end-stage sepsis or irreversible organ failure, the goal of microbiome modulation shifts from curative to symptomatic (60). Interventions may aim to reduce systemic inflammation, alleviate fatigue or fever, or lower the burden of pathogenic organisms to minimize recurrent infections and antibiotic burdens (61). The emphasis is on balancing intervention intensity with patient comfort, prioritizing quality of life and symptom relief within a patient-centered care framework (62).

### 4.4 Microbiome-informed decision support in palliative sepsis care

Within palliative care contexts, precision medicine shifts its primary objective from maximizing survival to supporting clinical decision-making under conditions of advanced illness, prognostic uncertainty, and complex care goal alignment (63). In this framework, microbiome-derived biomarkers offer an additional biological dimension to guide individualized, goal-concordant sepsis management strategies.

As illustrated in Figure 1, microbiome-derived signals may further contribute to identifying patients with limited physiological reserve and a reduced likelihood of recovery (16, 48). In particular, severe and persistent dysbiosis may function as a biologically plausible indicator supporting the reassessment of treatment goals, including consideration of a transition from life-prolonging interventions toward comfort-focused care (16). Importantly, such signals should not be interpreted in isolation but rather contextualized within a broader clinical, prognostic, and ethical framework.

Notably, no clinical trials to date have directly evaluated microbiome-guided decision-making in sepsis, particularly within palliative care settings (19). Existing applications are therefore predominantly inferential, grounded in observed associations between microbial patterns and clinical outcomes rather than validated interventional evidence (18, 64). This evidence gap underscores the current exploratory nature of microbiome-informed strategies and highlights the need for cautious interpretation (65).

Accordingly, direct clinical evidence supporting the use of microbiome-derived biomarkers to inform treatment decisions in sepsis—such as escalation or de-escalation of therapy, particularly in palliative care settings—remains limited (18). Current applications are largely exploratory and hypothesis-generating, underscoring the need for rigorously designed prospective studies to establish their clinical validity and real-world utility.

From a translational perspective, microbiome profiles may nevertheless provide meaningful adjunctive signals when integrated with established clinical parameters. Profound and sustained gut dysbiosis—characterized by markedly reduced microbial diversity, profound and sustained dysbiosis—may signal irreversible immunometabolic dysregulation rather than transient stress adaptation (30). For such patients, microbiome profiling could complement traditional clinical indicators to identify those with limited potential to benefit from further aggressive interventions, thereby informing timely transitions toward comfort-focused care pathways (66).

Conversely, relative preservation or early recovery of microbial diversity may indicate residual host resilience, supporting the continued use of targeted disease-modifying therapies in carefully selected patients (67). It is essential to emphasize that microbiome-informed stratification should be interpreted not as deterministic prognostication, but as probabilistic decision support, to be integrated holistically with clinical expertise, patient values, and family input (67).

Beyond prognostic stratification, microbiome insights may also inform symptom management in palliative sepsis (35). Dysbiosis-associated systemic inflammation has been linked to key symptoms such as fatigue, anorexia, fever, and recurrent infections, all of which contribute significantly to the symptom burden in advanced illness (68). Therefore, carefully designed and tolerable microbiome-modulating interventions may help alleviate symptoms, reduce unnecessary antimicrobial exposure, and improve quality of life in appropriate palliative care settings (18).

## 5 Challenges and future directions

### 5.1 Technical standardization and translational bottlenecks

Despite its promise in sepsis, translating gut microbiome research into clinical practice faces significant hurdles. A primary challenge is the lack of technical standardization across study protocols, from inconsistent sample collection and processing to variable sequencing and bioinformatic methods (69). These discrepancies limit the comparability and reproducibility of findings. Furthermore, most existing data demonstrate association rather than causation (70). Distinguishing whether microbiome alterations drive sepsis progression, result from it, or are influenced by treatments remains a critical methodological obstacle (71). Establishing robust causal inference is essential for advancing microbiome biomarkers from research tools to validated clinical diagnostics (72).

Beyond these methodological and technical constraints, the real-world implementation of microbiome-based approaches is further complicated by systemic and structural barriers across healthcare settings (73). High-throughput multi-omics technologies remain resource-intensive, requiring specialized infrastructure, advanced computational support, and interdisciplinary expertise (74). Such requirements may limit accessibility in low- and middle-income regions, thereby raising important concerns regarding health equity and the potential for unequal adoption of precision medicine strategies (75). In addition, current delays in data processing and interpretation restrict the feasibility of integrating microbiome profiling into time-sensitive clinical decision-making, particularly in critically ill patients with sepsis (76). Addressing these challenges will require the development of simplified, rapid, and cost-effective assays, alongside standardized workflows and scalable digital infrastructures, to enable broader and more equitable clinical implementation.

Importantly, these technical and implementation challenges are further compounded by overarching methodological limitations within the current evidence base. Much of the existing literature relies on observational study designs, often involving small sample sizes and heterogeneous patient populations, which collectively constrain statistical power and external validity (38). In addition, the absence of robust prospective validation and interventional trials limits the ability to establish causal relationships and to translate microbiome-derived findings into clinically actionable strategies (77). Collectively, these limitations represent critical barriers to the reliable integration of microbiome science into precision sepsis care. Addressing these gaps through large-scale, well-designed prospective studies, standardized methodologies, and rigorous validation frameworks will be essential to enhance both the robustness and the clinical applicability of future research.

### 5.2 Inter-individual variability and dynamic monitoring

High baseline variability between individuals complicates the definition of universal diagnostic thresholds (78). Future efforts should focus on establishing personalized microbial baselines through longitudinal profiling during stable health (79). This would enable detection of clinically meaningful deviations during critical illness. To support this, there is a need for rapid, cost-effective profiling methods suitable for near-patient testing (80). Real-time monitoring of microbiome dynamics in critically ill patients could identify actionable shifts aligned with clinical changes, paving the way for timely, personalized interventions (81).

### 5.3 Intervention safety and ethical considerations

The safety profile of microbiome-based therapies necessitates rigorous evaluation, particularly in vulnerable populations such as the immunocompromised and critically ill (82). Modalities like next-generation probiotics and FMT, while promising, carry inherent risks including pathogen transmission, systemic infection, and unintended ecological shifts within the microbial community (83). In the context of palliative care, these safety considerations are intrinsically linked to a broader ethical imperative (84). Any intervention should be carefully evaluated for its alignment with overarching patient-centered goals, which typically prioritize symptom relief, comfort, and quality of life over aggressive disease modification. Establishing a clear, transparent risk–benefit framework and ensuring a robust process of informed consent are therefore fundamental to the responsible development and clinical application of these therapies.

From a palliative care perspective, the ethical use of microbiome-based precision medicine extends beyond physical safety to encompass the management of prognostic uncertainty and the facilitation of shared decision-making (85, 86). Insights derived from the microbiome can serve as valuable tools for clinicians to explain disease trajectories more clearly, thereby supporting nuanced discussions about prognosis, therapeutic appropriateness, and the alignment of interventions with patient-defined values and care goals (87). Crucially, such biomarkers should be positioned as tools to inform—not to supplant—clinical judgment and the patient-clinician dialog (87). This approach ensures that biological data enhance and individualize palliative care planning without dictating it, preserving the primacy of holistic patient assessment and goal-concordant care (84).

### 5.4 Interdisciplinary collaborative framework

Progress depends on integrated collaboration across disciplines (88). Microbiologists, intensivists, palliative care specialists, bioinformaticians, and data scientists should work together to standardize research protocols, develop integrated analytical platforms, and design feasible clinical trials (72, 89, 90). Such synergy will be crucial for translating mechanistic insights into clinically useful tools and improving outcomes in sepsis through personalized microbiome science.

## 6 Summary

The gut microbiome represents a crucial new dimension in the precision management of sepsis. Its dynamic alterations prior to clinical onset, during acute illness, and throughout convalescence provide not only promising biomarkers for early risk stratification and prognosis but also reveal important insights into the disease’s underlying heterogeneity. Advancing beyond correlation toward causation, microbiome science is shifting the therapeutic paradigm from passive response to targeted modulation.

Integrating multi-omics data—including microbiomic, metabolomic, and clinical information—can refine sepsis management through a systems-level understanding of host-microbiome interactions. This approach holds particular promise in complex care settings such as intensive care. Furthermore, within palliative care, microbiome-informed precision approaches can support biologically grounded decision-making. Such approaches prioritize symptom relief, treatment proportionality, and quality of life instead of uniformly escalating care, thereby enabling more individualized and clinically nuanced decision-making.

To realize this potential, large-scale prospective cohort studies are urgently needed. These studies should implement standardized microbiome profiling protocols and rigorously validate their clinical utility in guiding decision-making. Ultimately, this work aims to establish an evidence-based, precision management pathway for sepsis that incorporates microbiome data, with the goal of improving patient outcomes and advancing critical care toward a more individualized approach.

## Data Availability

The original contributions presented in the study are included in the article/supplementary material, further inquiries can be directed to the corresponding author.
